# Proteomic Analysis of Honeybee (*Apis mellifera* L.) Pupae Head Development

**DOI:** 10.1371/journal.pone.0020428

**Published:** 2011-05-26

**Authors:** Aijuan Zheng, Jianke Li, Desalegn Begna, Yu Fang, Mao Feng, Feifei Song

**Affiliations:** 1 Key Laboratory of Pollinating Insect Biology, Ministry of Agriculture/Institute of Apicultural Research, Chinese Academy of Agricultural Science, Beijing, China; 2 Feed Research Institute, Chinese Academy of Agricultural Science, Beijing, China; Stockholm University, Sweden

## Abstract

The honeybee pupae development influences its future adult condition as well as honey and royal jelly productions. However, the molecular mechanism that regulates honeybee pupae head metamorphosis is still poorly understood. To further our understand of the associated molecular mechanism, we investigated the protein change of the honeybee pupae head at 5 time-points using 2-D electrophoresis, mass spectrometry, bioinformatics, quantitative real-time polymerase chain reaction and Western blot analysis. Accordingly, 58 protein spots altered their expression across the 5 time points (13–20 days), of which 36 proteins involved in the head organogenesis were upregulated during early stages (13–17 days). However, 22 proteins involved in regulating the pupae head neuron and gland development were upregulated at later developmental stages (19–20 days). Also, the functional enrichment analysis further suggests that proteins related to carbohydrate metabolism and energy production, development, cytoskeleton and protein folding were highly involved in the generation of organs and development of honeybee pupal head. Furthermore, the constructed protein interaction network predicted 33 proteins acting as key nodes of honeybee pupae head growth of which 9 and 4 proteins were validated at gene and protein levels, respectively. In this study, we uncovered potential protein species involved in the formation of honeybee pupae head development along with their specific temporal requirements. This first proteomic result allows deeper understanding of the proteome profile changes during honeybee pupae head development and provides important potential candidate proteins for future reverse genetic research on honeybee pupae head development to improve the performance of related organs.

## Introduction

The honeybee displays complete metamorphosis where an individual passes through 4 developmental stages: egg, larva, pupae and adulthood. After 3 days, the egg hatches into a feeding stage called the larva. Six days later, the larva enters an intermediate and inactive stage known as the prepupa sealed in a cell by beeswax. After a few hours, internal changes begin that transform the prepupa into a quiescent white pupa with 3 major body regions that superficially look like those of an adult bee. With gradual darkening, hairs and wings develop and after a few days an adult bee emerges by chewing out of the wax cell capping [1], [2]. The pupa stage is the longest post embryonic developmental period of the honeybee. The temperature at which pupae are raised influences the tasks and behavioral determination of the adult bees [3]. It is reported that pupae weight increases with honey production and pupae head weight increases with higher royal jelly production [4]. Comparative biochemical analysis between worker and queen heads has revealed that adults raised under higher temperatures show higher probability to dance, forage earlier, and more often are involved in more activities [3], [5]. Ecdysteroid titer production levels peak earlier in queen than worker pupae [6]. The head of honeybee is formed at the pupa stage and consists of the brain and associated ganglia, hypopharyngeal glands (HGs), mandibular glands, salivary glands, and antennae, which contribute to neural, endocrine and/or exocrine functions [7]. The head of the adult honeybee has been widely investigated morphologically [8], [9], biochemically [10] and molecularly [11]–[15]. However, proteomic studies on honeybee pupae at different developmental stages and even on sub-organs are very limited. Recent completion of the honeybee (*Apis mellifera* L.) genome sequence [16] has opened promising ways for detailed investigations of honeybees using the proteomic approach. In this study we investigate honeybee pupae head protein expression profile changes at different developmental stages using the proteomic approach to gain better understanding of molecular factors involving in shaping the pupae head development.

## Materials and Methods

### Chemical Reagents

The following reagents were purchased: Urea, Tris-base, sodium dodecyl sulfate (SDS), sodium bicarbonate, dithiothreitol (DTT), iodoacetamide and bovine serum albumin (BSA) were purchased from Sigma (St. Louis, MO, USA). Bio-lyte from Bio-Rad (Hercules, CA, USA), acrylamide, N, N′-methylenebisacrylamide, ammonium persulfate (AP), N,N,N′,N′-tetramethylethylene diamine (TEMED), 3-[(3-cholamidopropyl) -dimethylammonio]-1-propane sulfonate (CHAPS), glycerol, bromophenol blue, flamingo fluorescent dye (Bio-Rad), coomassie brilliant blue (CBB) G-250. α-cyano-4-hydroxycinnamic acid (CHCA) from Bruker Daltonics (Billerica, Mass. USA), trypsin from Roche (Modified, Sequencing Grade, Roche, Mannheim, Germany), and trifluoroacetic acid (TFA) and acetonitrile from J. T. Baker (Phillipsburg, NJ, USA). All the chemicals used for RNA isolation and real-time PCR were purchased from Bio-Rad (Hercules, CA, USA). Other chemicals used, but not specified here are noted in the text.

### Biological Samples

**Figure 1:**
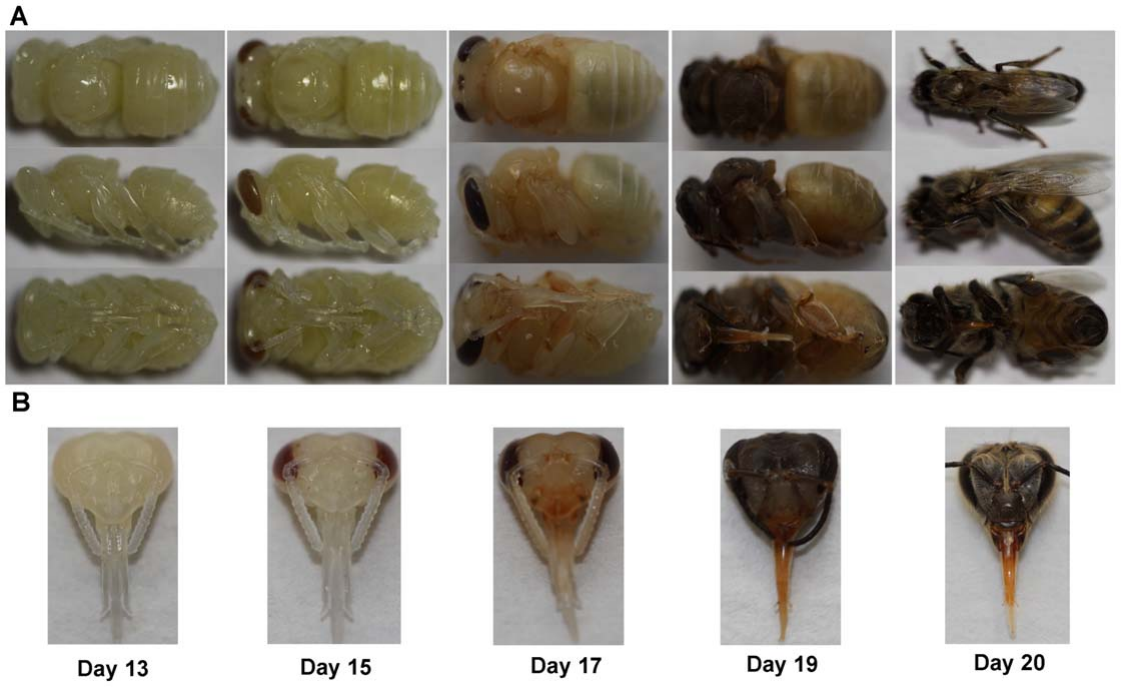
Image of honeybee pupae in different positions at different developmental stages showing the occurrences of gradual changes in phenotype and organogenesis as time passes. (A) and (B) represent corresponding whole pupal body and head development.

Honeybee (*Apis.mellifera lingustica*) worker pupae were collected on day 13, 15, 17, 19 and 20 from the apiary of Institute of Apicultural Research, Chinese Academy of Agricultural Science in May 2009. The exact age of the pupae were obtained by confining the egg laying queen for 5 hours on a single wax comb frame containing worker cells using a queen excluder. Subsequently, the queen was removed and the frame containing the eggs was maintained in the honeybee colony until the date of collection. A total of 100 pupae heads were sampled for each time point based on the compound eye pigmentation (Fig. 1) from 5 bee colonies having similar conditions sourced and the heads were dissected and stored at −80°C until analysis.

### Protein Extraction and Two-dimensional Gel Electrophoresis (2-DE)

Protein extraction was carried out as described previously [17]. Protein quantification was performed according to the method developed by Bradford [18] using BSA as the standard and the absorption was measured at 595 nm (Beckman, spectrophotometer DU800).

A 450 µg protein sample was suspended in lysis buffer [8 M urea, 2 M thiourea, 4% CHAPS, 20 mM Tris-base, 30 mM DTT, 2% Bio-lyte pH 3–10] and then mixed with rehydration buffer [8 M urea, 2% CHAPS, 0.001% bromophenol blue, 45 mM DTT, 0.2% Bio-lyte pH 3–10]. The mixture was loaded on a 17 cm IPG strip (pH 3–10, linear, Bio-Rad). Isoelectric focusing (IEF) was performed at 18°C according to manufacturer's instructions (Protean IEF Cell, Bio-Rad). Before SDS-PAGE, the IPG strips were first equilibrated for 15 minutes in equilibration buffer 1 [6 M urea, 0.375 M Tris-HCl (pH 8.8), 20% glycerol, 2% SDS, 2% DTT] and then continued in equilibration buffer 2 [6 M urea, 0.375 M Tris-HCl (pH 8.8), 20% glycerol, 2% SDS, 2.5% iodoacetoamide] for another 15 minutes. Second dimension electrophoresis, SDS-PAGE, was performed in a Protean II Xi Cell (Bio-Rad) at 25 mA/gel for 6 hours.

### Image Acquisition and Statistics Analysis

Gels were fixed overnight in 50% (v/v) ethanol with 10% (v/v) acetic acid, washed in water, and stained with Flamingo fluorescent dye (Bio-Rad) for image analysis and then further dyed with CBB G-250 to visualize spots for mass spectrometry (MS) analysis. Three independent biological replicates 2-DE gel images were digitized with ImageScanner III (GE Healthcare) at 16 bit and 300 dpi resolution. Image filtration, background subtraction, spot detection, spot matching, and quantitative intensity (all the pixels making up the spot) analysis were performed using PDQuest software (ver. 8.0.1, Bio-Rad). All gels were matched with one of the selected reference gel. The match analysis was performed in an automatic mode, and further manual editing was performed to correct the mismatched and unmatched spots. The expression level of a given protein spot was expressed in terms of volume of the spot. To compare spot quantities between gels accurately, the spot volumes were normalized as percentage of the total volume of all of the spots in the gel. The means and standard deviations from the triplicate experiments were calculated and the statistical significance of the expression level of the protein and mRNA at differential time-stage were assessed with one-way ANOVA (SPSS Version 16.0, SPSS Inc.), a Duncan's Multiple Range test was used to compare the difference between means of the expression level at 5 time-point. An error probability of *p*<0.05 was considered to be statistically significance of at least 1.5 fold changes.

### Identification of Differentially Expressed Protein

Proteins showing significant expression were excised and denatured, alkylated, trypsin digested as described previously [11]. Matrix was prepared by dissolving α-cyano-4-hydroxycinnamic acid (CHCA, Bruker Daltonics) in 50% acetonitrile/0.1% trifluoroacetic acid. Ten microliters of solution was added onto the dried digests and vortexed for 30 min. A total of 1.5 µL of the reconstituted in-gel digest sample was spotted initially on Anchorchip target plate (600/384F, Bruker Daltonics), followed by 0.5 µL of matrix solution. The dried sample on the target was washed twice with 1 µL of 0.1% TFA, left for 30 seconds before solvent removal. The digested peptide mixture was analyzed by an Ultraflex II matrix assisted laser desorption ionization time of flight/mass spectrometry mass spectrometer (MALDI-TOF MS) (Bruker Daltonics) under the control of Flex Control 2.2 software (Bruker Daltonics). MALDI-TOF spectra were recorded in the positive ion reflector mode in a mass range from 700–4000 Da and the ion acceleration voltage was 25 kV. Acquired mass spectra were processed using the software Mascot distiller (Version 2.2, default settings, Matrix Science) by default setting. Spectra were calibrated by a protonated mass signal from a standard peptide calibration mixture consisting of 8 peptides covering mass range from 700 to 3100 with (Bruker, Billerica, MA Peptide Calibration Standard 206196). Spectra originating from parallel protein digestions were compared pairwise to discard common peaks derived from trypsin autodigestion or from contamination with keratins. The resulting peptide mass lists were used to search against the nonredundant NCBI (NCBInr, release date, January 22, 2010) using MASCOT 2.2 (Matrix Science). Search parameters were: Taxonomy: Apis mellifera; trypsin cleavage; allow up to one missed cleavage; peptide mass tolerance 0.2 Da; fixed modification: carbamidomethyl (C); variable modification: oxidation (M). A total of 10,348,164 sequences and 3,529,470,745 residues in the database were actually searched.

When the identified peptides match to multiple members of a protein family, or a protein appears under the same names and accession number, the match was considered in terms of higher Mascot score the putative function and differential patterns of protein spots on 2-DE gels. Protein identifications were accepted if the established probability was greater than 95% and contained at least 2 identified peptides having maximum peptides coverage.

### Bioinformatic Analysis

The expression profiles were performed using expression values of protein spots at different developmental time point by calculating average distances using cluster software (Gene cluster, version 3.0).

Biological interaction networks (BIN) of the differentially expressed proteins were analyzed using Pathway Studio. Hence, experimental results were interpreted based on the context of pathways, protein regulation networks, and protein interaction maps in the *Drosophila* molecular networks database, which is equipped with functional relationships from other scientific literature. The applied filters included “all shortest paths between selected entities.” The information received was narrowed down to our protein list of interest, namely, those proteins whose involvement and regulatory functions had been observed. Each link was built with evidence from at least 3 publications. The interactions between the imported proteins and all proteins stored in the database were then identified. Protein entities which belong to different functional groups were represented as different shapes according to the default settings of the software as shown in the legend.

To enrich the identified proteins to specific functional terms, the protein lists were analyzed using the ClueGo software and applying the *Drosophila* database from the Gene Ontology database (release date, January 10, 2010). Ontology selection as biological process and enrichment analysis was done by right-side hyper-geometric statistic test and its probability value was corrected by the bonferroni method [19].

### Validation of Differentially Expressed Proteins

To further verify the differential expression levels, quantitative real-time PCR was run for proteins identified at day 13, 15, 17, 19 and 20. Based on the results of the gel-based comparison, specific primers (Table S1) suited to simultaneously amplify various target genes were designed according to the corresponding gene sequences of the identified proteins and the available gene information in the GenBank library by using the primer design software (Beacon Designer 7.51, PREMIER Biosoft International Palo Alto, CA). Total RNAs were prepared from the head on day 13, 15, 17, 19 and 20 using TRIzol reagent (Takara bio) and cDNA synthesis was performed using TaKaRa RNA PCR Kit (AMV) Ver.3.0 (Takara bio), according to the manufacturer's instruction. Real-time PCR was conducted using an iQ5 Multicolor Real-Time PCR Detection System (Bio-Rad). PCR was performed in 25-µl reaction system containing 1 µl cDNA, 5 pmol forward and reverse primers, 12.5 µl SYBR Green Supermix (Bio-Rad) and water. Fold-change was calculated using the 2^−ΔΔCt^ method [20].

For Western blot, each of ald (spot d17), hsp60 (spot d9), Tcp-1η (spot d13) and idh (spot u9) were subjected to 3 replication runs and 4 µg of protein samples were loaded on each lane separated by stacking (4%) and separating (12%) SDS-PAGE gels. To ensure that the specific anti-ald, hsp60, Tcp-1η and Idh bands could be detected, protein molecular marker was loaded when running the gels. Gels were run at a voltage of 120 V for approximately 1.5 h using Mini-Protein II Gel electrophoresis system (Bio-Rad Laboratories Ltd.). Resolved proteins were transferred to a nitrocellulose (NC) transfer membrane (0.2 µm pore size) (Invitrogen) using the iBlot apparatus (Invitrogen, Carlsbad, CA). Nonspecific binding was blocked with 5% (w/v) nonfat milk powder in the Tris buffered saline (20 mM Tris-HCl, 150 mM NaCl, pH 7.6) containing 0.1% (v/v) Tween-20 (TBS-T) at room temperature for 1 h. The membranes were then incubated with primary rabbit polyclonal anti-ald, hsp60, Tcp-1η and Idh antibodies (Abcam, Massachusetts) at a dilution of 1∶5000 in 2% milk powder in TBS-T at 4°C overnight. Following three washes, the membranes were further incubated with goat anti-rabbit IgG conjugated with horseradish peroxidase (Pierce, Rochford, IL) (1∶10,000 in 2% milk powder in TBS-T), and were rolled for 1.5 h at room temperature. At the end of this process, the NC membranes were washed for 2 h, rolled at room temperature. Immunoreactive protein bands were then visualized by enhanced chemiluminescence detection (ECL, Pierce, Rochford, IL) reagents and quantified by densitometry using the Quantity-one image analysis system (Bio-Rad Laboratories Ltd.). The human anti-β-actin antibody (1∶ 5000, sigma) was detected simultaneously as a loading control.

## Results

**Figure 2:**
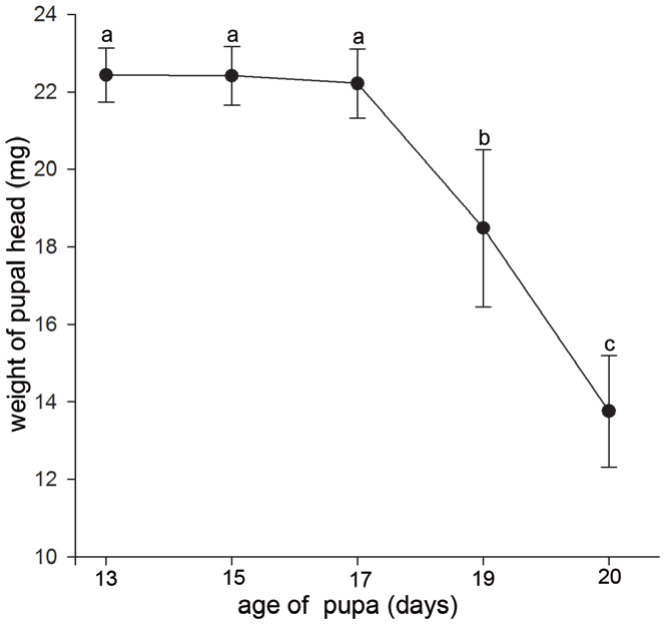
Pattern of honeybee pupae head weight at different developmental time points. Different letters (a, b, c) are significantly different (p<0.05).

**Figure 3:**
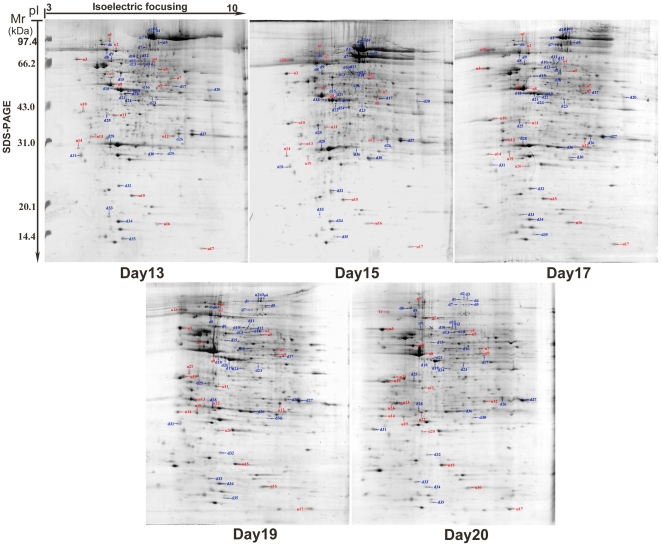
2-DE and CBB G-250 stained gels image showing differentially expressed protein spots profile obtained from different developmental stages of honeybee pupae heads. Each identified protein spot is specified by number and indicated by arrow, where the spot number with prefix “u” and “d” indicate up or down regulation.

In order to understand the developmental progresses, pictures and weights of the pupae head were taken at 5 time-points (Fig. 1). Accordingly, the pupae head weight between 13–17 days was significantly heavier than those at 19–20 days (Fig. 2). Subsequently protein extracts from pupae head were separated using 2-DE and comparisons were done across 5 time points. As a result, the molecular weight (*Mr*) and p*I* of pupae head proteins ranged from 10.45 to 125.16 kDa and 3.7 to 9.56, respectively (Fig. 3). The 2-DE image analysis revealed nearly 400 spots on each gel, of which 85 spots showed differential expressions (>1.5 fold change, *p*<0.05) and 59 of them were successfully identified by MALDI-TOF MS (Table S2 and Fig. 3). The remaining unidentified proteins spots could be attributed to their lower abundance to produce enough spectra, or because the databases search scores were not high enough (<95%) to yield unambiguous results. In this study, some protein spots were identified as the same proteins, but appeared differently on 2-DE gels, most likely due to post-translational modifications, such as phosphorylation and possibly alternative splicing or proteolysis that results in shifting of *M*r and p*I*.

### Functional Classification of the Identified Proteins

**Figure 4:**
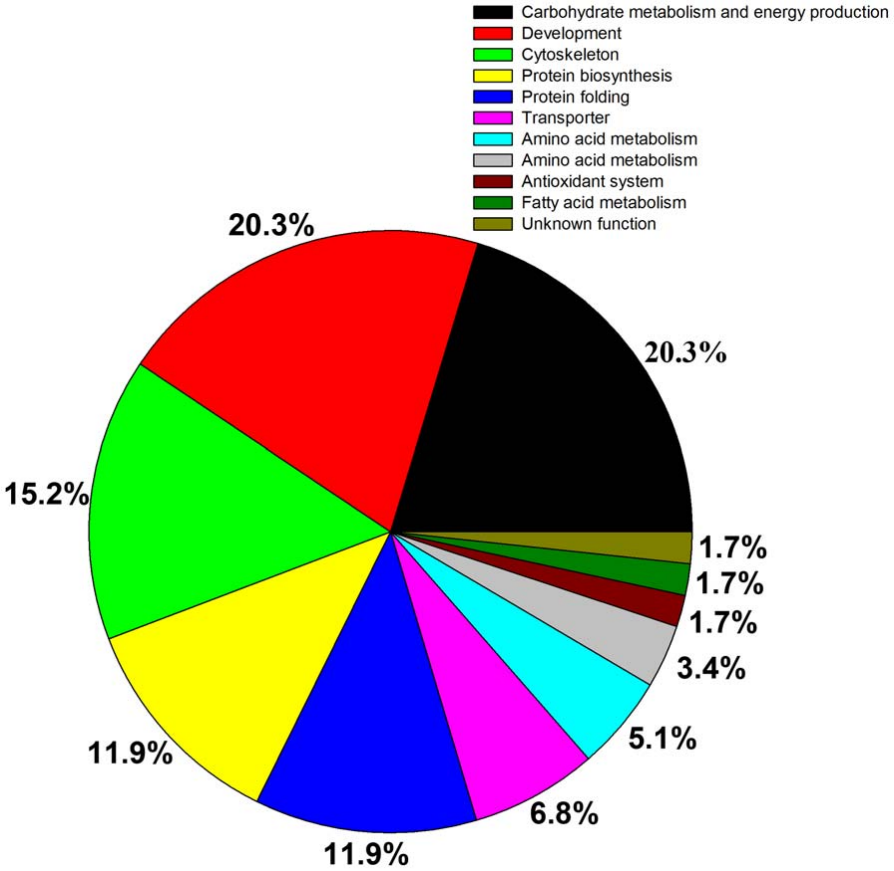
Functional annotation and distributions of the differentially expressed proteins identified from honeybee pupae head at different developmental time points.

**Figure 5:**
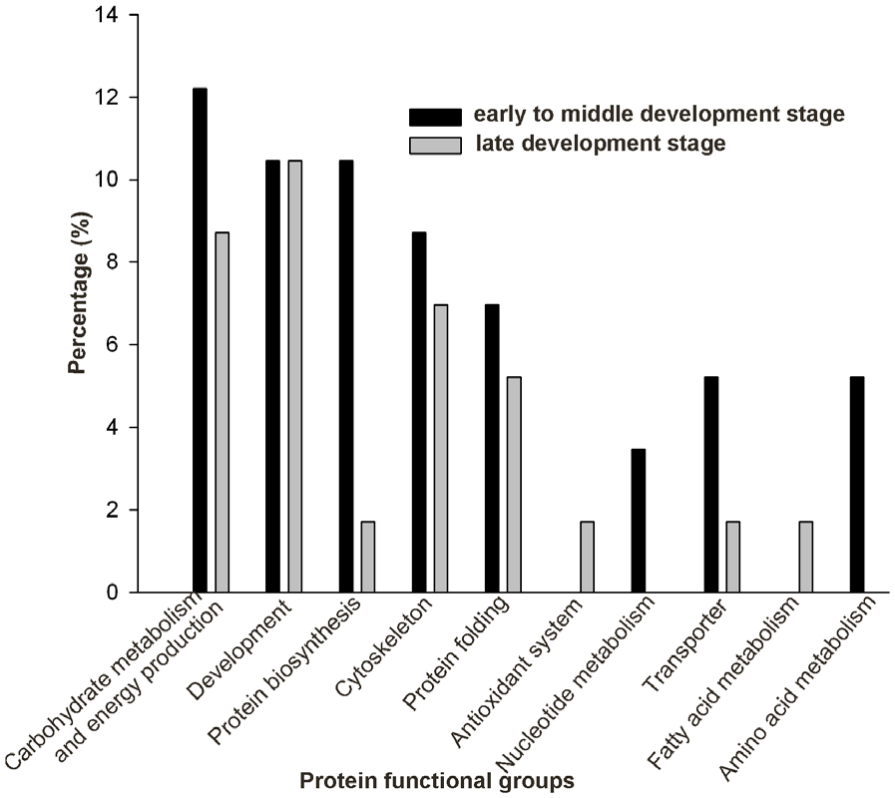
Functional comparisons of upregulated proteins between the early (13–17 days) and late (19–20 days) stage of honeybee pupae head.

The identified proteins were grouped into 11 functional classes and proteins involved in carbohydrates metabolism and energy production, development, cytoskeleton, protein folding and protein biosythesis were found to be the major protein families (Fig. 4). Interestingly, the proportions of functional classes (except antioxidant and fatty acid metabolism proteins) indicated higher representation from early to middle than the late developmental stages. Specifically, nucleotide and amino acid metabolisms proteins were expressed only during the early to mid developmental stages (Fig. 5).

### Hierarchical Cluster Analysis of Differentially Expressed Proteins

**Figure 6:**
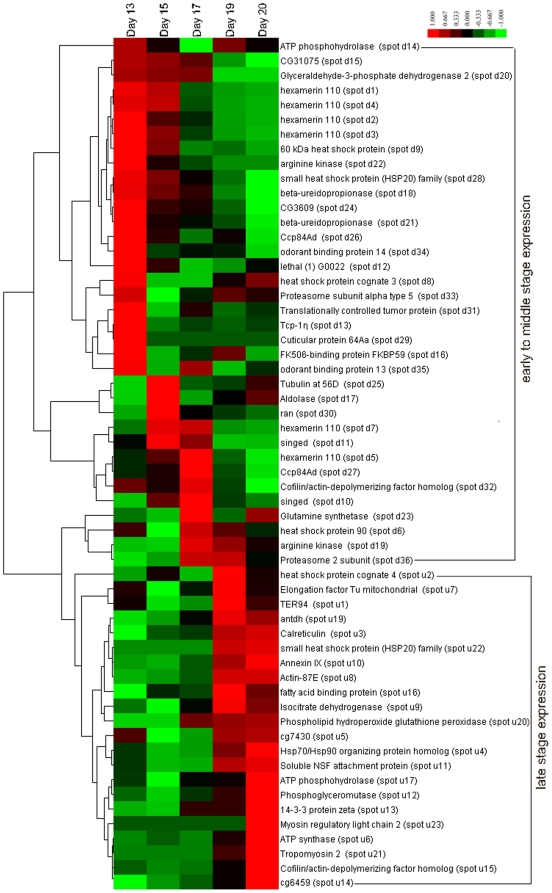
Global expression intensity map (hierarchical clustering) showing upregulation (red) and downregulation (green) across the ages of the pupae indicated on the top of each column with lists of functional proteins in the right column.

The hierarchical cluster analysis of differentially expressed proteins showed that 58 distinct proteins (excluding 1 protein with unknown functions) were partaken in the expression intensity map (Fig. 6). Generally, most of the protein spots behaved heterogeneously, but clustered under 2 large very homogenous expressional pictures that were from the early to middle stages (day 13–17) and the late developmental stage (day 19–20) with a shifting trend inline with the pupae head developmental stage. From 36 protein spots that were highly expressed during the early to middle stages, proteins as carbohydrate metabolism and energy production, development, protein biosythesis, cytoskeleton and protein folding were recognized as major groups (Fig. 6). To this fact, there were 7 proteins spots involved in carbohydrate metabolism and energy production (spots d14, d15, d17, d19, d20, d22 and d24), 6 in development (spots d10, d11, d12, d13, d30 and d31), 6 in biosythesis (spot d1–4, d5, d7), 5 in cytoskeleton (spots d25, d26, d27, d29 and d32) and 4 in protein folding (spots d6, d8, d9 and d28). In addition, there were 3 amino acid metabolism (spots d23, d36 and d33), 3 molecular transporters (spots d16, d34 and d35) and 2 nucleotide metabolism (spots d18, d21) that were upregulated during the early to middle stage. On the other hand, 22 proteins were upregulated during the late pupae stage that included 6 in development (spots u1, u3, u10, u11, u13 and u14), 5 in carbohydrate metabolism and energy production (spots u5, u6, u9, u12, and u17), 4 in cytoskeleton (spots u8, u15, u21 and u23) and 3 in proteins folding (spots u2, u4 and u22). The other proteins upregulated during late developmental stages included one involved in molecular transporter (spot u19); one related to protein biosynthesis (spot u7), one associated with fatty acid metabolism (spot u16) and one involved in antioxidant activity protein (spot u20) (Fig. 6).

### Functional Enrichment Analysis

**Figure 7:**
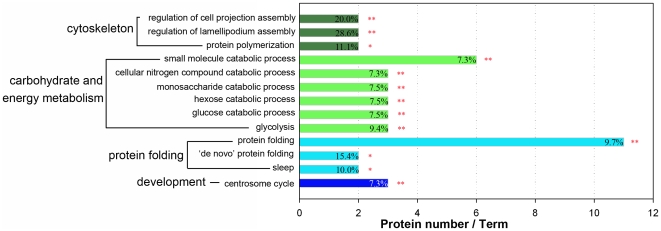
Functional enrichment analysis of the differentially expressed proteins using the ClueGO software.

Gene Ontology (GO) annotation provides 3 detailed and structured terms that include molecular functions, biological processes, and cellular components, which are currently widely used in the analysis of large proteomic and genomic datasets. Significantly overrepresented GO terms are examined to determine hypotheses for the biological events behind the data and assist in providing a broad overview of the principal characteristics of a proteome. Functional enrichment analysis was conducted using the ClueGo software on all proteins that had altered their expression across the 5 time-points. Hence, carbohydrate metabolism and energy production, protein folding and cytoskeleton, development related proteins were significantly enriched (Fig. 7).

### Network Analysis

**Figure 8:**
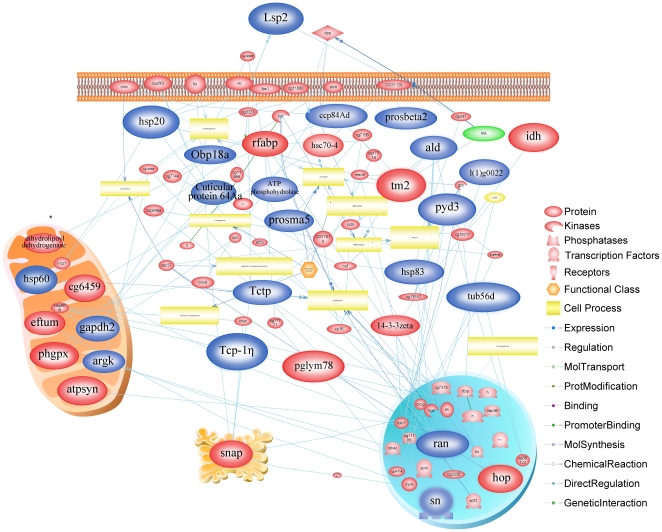
Biological interaction network of the identified differentially expressed proteins from honeybee pupae head at the different developmental stages. The big ellipse represents identified proteins in this experiment and each entity belongs to the category described in the legend. The “Blue” and “Red” are the proteins upregulated during early to middle development stages (13–17 days) and during late developmental stages (19–20 days), respectively.

Pathway Studio analysis was employed to predict the biological interaction network (BIN) with interactive relations for the differentially expressed proteins. Accordingly, 33 proteins were recognized as key nodes with various relations in the created BIN. The result from the established network clearly indicated that carbohydrate metabolism and energy production, development, protein folding and cytoskeleton were the most linked protein families and protein categories and as can be seen in Fig. 8 the upregulated species are color coded in the legend.

### Validation of Differentially Expressed Proteins

**Figure 9:**
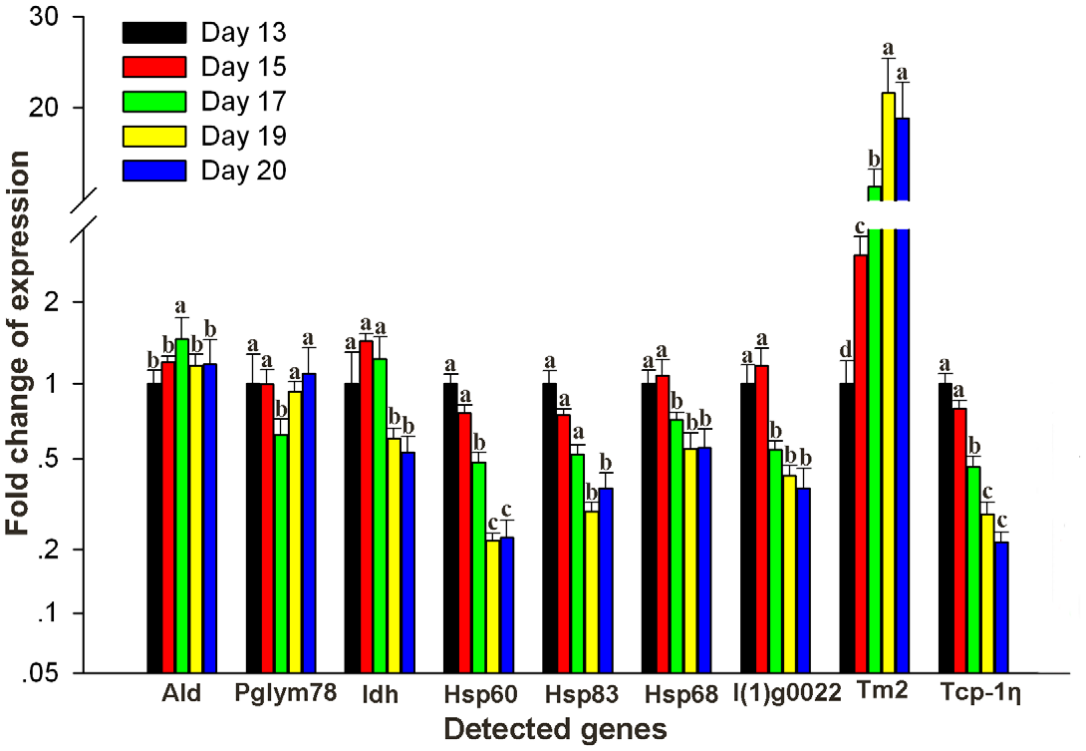
Transcript validation of differentially expressed proteins at different development periods. mRNA of different development days are measured by quantitative real time PCR. Samples are normalized with reference gene (GAPDH) and with the expression abundant on day 13 as the reference sample. Error bar is standard deviation. Gene symbols indicating different genes refer to Table S2. Different letters (a, b, c) are significantly different (p<0.05).

**Figure 10:**
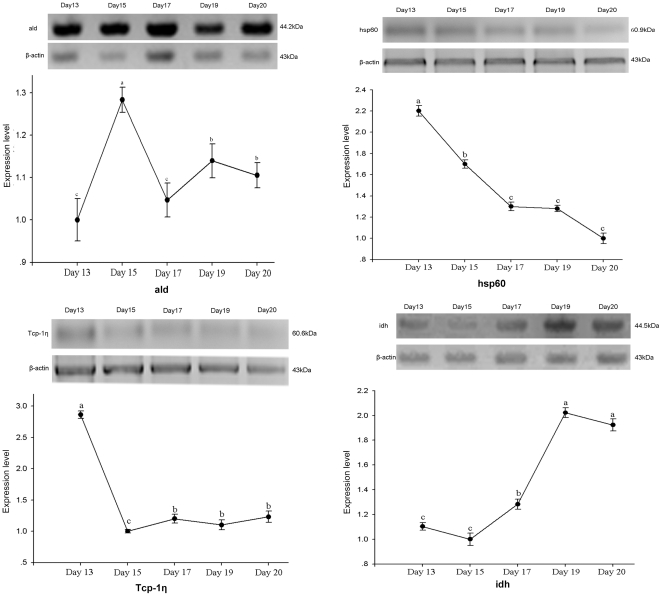
Western blot analysis of ald, hsp60, Tcp-1η and idh. y-axis represents relative expression level normalized by β-actin, x-axis represents different development stages on day 13, 15, 17, 19 and 20, accordingly. Different letters (a, b, c) are significantly different (p<0.05).

Among the 4 major groups (carbohydrate and energy production, protein folding, development and cytoskeleton) involved as key nodes in the BIN, 9 proteins were selected for further validating the proteins differential expressions at mRNA levels (Fig. 9) of which 4 of them were further confirmed by Western blot analysis (Fig. 10). The selected proteins were, ald (spot d17), pglym78 (spot u12), idh (spot u9), hsp60 (spot d9), hsp83 (spot d6), hsc70-4 (spot u2), l(1)g0022 (spot d12), Tcp-1η (spot d13) and tm2 (spot u21). Accordingly, 6 proteins showed consistent mRNA expressions with the change patterns of their corresponding proteins as in 2-DE gels. The 6 proteins that showed consistent increased mRNA abundance were ald (spot d17), hsp60 (spot d9), hsp90 (spot d6), l(1)g0022 (spot d12), tm2 (spot u21) and Tcp-1η (spot d13) and they were gene transcripts from early to middle stage (Fig. 9). However, the transcripts of genes of pglym (spot u12), idh (spot u9) and hsc70-4 (spot u2) showed variations between the mRNA transcription and protein abundance across the 5 time points and this might be due to lack of a direct relationship between mRNA timing and protein expressions and/or other regulatory mechanisms such as lack of synchronization. The result of the Western blot analysis also showed considerable expressional difference for ald (spot d17), hsp60 (spot d9), Tcp-1η (spot d13) and idh (spot u9) and the achieved differences were in line as in the 2-DE image (Fig. 10).

## Discussion

In the life of the honeybee pupal stage follows the larval stage and precedes adulthood. It is at the pupal stage that the adult structures are formed and the larval structures are broken down. The generation of organs inside the head mainly occurs at this stage and hence, vital proteins have to be involved to support their initiation, formation, and completion. Although it is known that increased pupae weight is correlated with honey [21], and pupae head weight with royal jelly production [4] these studies were limited in morphological and phenotypic investigations, which required a follow up study to uncover the molecular factors, which cause these phenotypic phenomenon to occur. Accordingly, our study reveals that proteins that are related to carbohydrate metabolism and energy production, development, cytoskeleton and protein folding are involved in accelerating the development and metabolism of honeybee pupae head development in general as well as salivary glands, hypopharyngeal glands and are involved in restructuring of nervous system development, in particular. Specifically, proteins involved in biosynthesis and metabolizing amino acid were upregulated in the pupal head during early stages of development (13–17 days) suggesting tremendous ongoing physiological processes to ensure the formation, development and structuring of important organs inside the pupae head. On the other hand, the role of those proteins species that upregulated during the late developmental stage (19–20 days) were found to ensure the neuron development and to activate the functional glands (Fig. 5, 6). Following this functional mission, most of the differentially expressed proteins showed a shift in species in line with developmental stages of the pupae head as a sign of task accomplishment.

The development of salivary glands will help in the production of enzymes, which will help with the honey ripening process (sugar breakdown). Furthermore, the nervous system restructuring could help in the development of the olfactory systems, which is important in pollen and nectar collection by the forager bees and ultimately contributes to improved honey production.

The significantly heavier weight of pupae heads during the period between 13 to 17 days was positively related to the high amount and varieties of upregulated proteins (36 of 58) suggesting the biological success of fast growing pupae head depends on the participations of high amounts and different varieties of proteins.

Carbohydrate metabolism and energy production proteins are well known to play a major role in the process of developing worker embryos, larvae and HGs [11], [17], [22]. Hence, the energy metabolism proteins are required as a key metabolic fuel by the worker bees for foraging flight [23] and as nutrients for neurons for learning and memorization processes [24], [25]. As well, their over expressions in honeybee pupae head at early developmental stage suggests high metabolic rates that demand high energy to ensure the formation of important organs, head growth, neuron development and metamorphosis. However, their typical late developmental stage over-expressions might suggest their crucial involvement in shaping and completion of full organ formation as well as to equip them with physiological functions, like capabilities of secreting royal jelly from HGs [11], acquire learning ability from neuron systems, odorant from olfactory systems and other basic tasks to be performed after emerging as an adult.

A group of development related proteins (spot d10, d11 and u14) that regulate the pupae head development as well as the development of bristle and hair in *Drosophila* [26], [27] were upregulated. Similarly, Tcp-1η (spot d13) and l(1)g0022 (spot d12) proteins have been known in mitotic spindle organization [28] and centriole replication [29]. Ran (spot d30) is involved in regulating cell shape, cell adhesion and cell cycle [30], [31]. Tctp (spot d31) positively regulates cell size and eye growth [32]. Hence, their elevated expressions from early to middle stage suggests their involvement in regulating cell size, growth, cell bond and cell cycle for the pupae head development as well as in the formations and development of basic organs and their functionalities. For instance, the nuclear encoded mitochondrial protein fork head (spot u14) is known to have a biological process of salivary gland development; ecdysone-mediated induction of salivary gland cell autophagic cell death; and salivary gland morphogenesis [33]. And the late developmental stage over-expressions of snap (spot u11) having a function of synaptic transmission and compound eye morphogenesis [34] suggests its vital role in ensuring the formation of functional compound eye. Also, ter94 (spot u1) has neuropil function in the mushroom body and antennal glomeruli of adult *Drosophila* head [35], crc (spot u3) with neuronal development, olfactory system and odor-guided behavioral functions in *Drosophila* and 14-3-3 zeta (spot u13) is known to assist in the process of learning and long-term memory formation of the honeybee [7]. In addition, the late stage over-expression of AnnIX (spot u10) is likely to control cell apoptosis in the HG through managing programmed cell death [36], [37].

The major role of the storage proteins is to serve as a reservoir for amino acids which will be utilized for tissue formation later during the adult development [38]. Recently, hexamerins have been reported acting as storage proteins for gonad development, egg production, and support foraging activity [39]. Therefore, the over-expressions of 6 forms of hexamerin 110 from the early to middle stage of pupae head in this study is in line with the investigations for *Drosophila* that showed increased expressions of storage proteins during 3rd larval instar to pupae stage [40], [41]. Thus, the heavier pupae head weight during the early to middle stage is likely because of the higher protein storage that was stored in the fat body of pupae and hence in the younger pupae [42].

Protein folding is the physical process by which a polypeptide folds from a random coil into a characteristic and functional three-dimensional structure, which is essential for its functionality [43]. The currently identified heat shock protein (hsps) is induced in honeybees either at high temperatures [44] or when the bee suffers from pathogen infections [7], [45]–[47]. However, because our experiment was carried out under normal circumstances, its upregulation serves only as molecular chaperones in living cells to ensure that cell proteins function correctly [48], [49] as was reported for worker embryos [17], HGs [11], larvae [22], workers brain [24], [50], hemolymph [51] and venom gland [52].

Cytoskeleton proteins have dynamic structures helping to maintain cells shape and play a vital role in both intracellular transport and cellular division. Alterations of several cytoskeletal protein expression as actin-binding, myosin, cuticle were reported in pupae heads after bacterial challenge [7]. Upregulations of actin 88F has been reported in the nurse bee brain in relation to the olfactory system [24] and in the normal embryogenesis of the worker bee and HGs [11], [17]. Therefore, the dynamic expressions of cytoskeleton proteins in this study suggests a significant role in providing skeletal element to maintain cell scaffold and to be involved in the processes of organ formations and to equip the organs with properly functioning facilities as in *Drosophila* [31], [53]–[56].

Proteasomal proteins play an essential role in the degradation pathway to supply amino acids for fresh protein synthesis. It was further reported that they are also involve inthe processes of honeybee HG gland development and royal jelly secretions [11]. In this study, the upregulations of 3 proteins related to the metabolism of amino acid (spot d23, d36 and d33) at the early developmental stage suggests their requirement as a nitrogen building block in the fast growing glands residing in honeybee pupae head.

Odorant-binding proteins (OBPs, spots d34, d35) are required for recognition of chemical stimuli in olfactory system of insects and reported in the forager honeybee antennae and larvae developmental process [57], [58]. The protein FKBP59 (spot d16) is known to regulate calcium ion transport and detect light stimulus in the visual perception of *Drosophila* [59]. The increased expressions of these molecular transporter proteins in the young pupae head indicate their vitalities as general carriers in the developmental and physiological processes [60]. However, the over-expressions of antdh (spot u19) in the old pupae head suggests its possible preparations for the olfactory roles associated with odor-based communication during the nurse bee stage development [24]. In addition, the differential expressions of proteins as rfabp (spot u16), antioxidant activity (spot u20) and pyd3 (spot d18, d21) were in line with findings for the developing honeybee larvae, embryos and HGs to metabolize fatty acid, nucleotides and for removal of damage by reactive oxygen species [22], [60], [61].

Since the completion of honeybee genome sequence, RNA interference has already been proved to be a successful tool for *in vivo* studies of gene function and phenotypes [62], [63]. The matching validation results between the protein and the genes provide information on the potential genes to manipulate honeybee at the gene level to increase better understanding on biological aspects and pave ways towards an effort to improve the performance of important organs like HGs, salivary glands and olfactory system residing in the head.

In conclusion, our global proteomic results provide an overview on the systemic honeybee pupae head development at the protein level. From the identified protein species and their amounts, it is clear that the development of young pupae head requires specific proteins to develop as well as to generate associated organs residing. Young pupae head involves more biosynthesis and metabolizing amino acids proteins than old pupae head do. The old pupae head involves proteins which are primarily related to the development and restructuring of organs like neuron and olfactory systems. The bioinformatic analysis also showed that proteins involved in metabolic energy, regulation of development, cytoskeleton and protein folding were the major contributors to pupae head development. Both the predicted biological network and the validation results helped us to detect important key node proteins for future target functional analysis. Thus, further study will be required to investigate what possible roles these proteins could play in support of honeybee pupal head development by RNAi, or by inhibiting the proteins' functions by pharmacological approaches (e.g. hsp, proteasome and cytoskeletal inhibitors). To our knowledge, this is the first proteomic report, which offers new insight into honeybee pupae head development and provides valuable information and expands our knowledge of the biology of honeybees.

## Supporting Information

Table S1 The primer sequences used for real-time PCR of the differentially expressed genes in honeybee (*Apis mellifera* L.) pupae head at different developmental stages.
(DOC)

Table S2 Identification of differentially expressed proteins in honeybee (*Apis mellifera* L.) pupae head at different developmental stages.
(DOC)
