# Supplementary material for: Proteomic Analysis of Honeybee (Apis mellifera L.) Pupae Head Development
Source: PLoS One. 2011 May 26;6(5):e20428. doi: 10.1371/journal.pone.0020428 (PMC3102718; doi:10.1371/journal.pone.0020428)
Supplement: Table S1 — The primer sequences used for real-time PCR of the differentially expressed genes in honeybee (Apis mellifera L.) pupae head at different developmental stages. (DOC) [file pone.0020428.s001.doc]

**Table S1.** The primer sequences used for real-time PCR of the differentially expressed genes in honeybee (*Apis mellifera* L.) pupae head at different developmental stages

| **Spot**  **No.** | **Abbreviation** | **Accession No.** | **Protein Name** | **Primer Sequence** | **Sense 5’-3’** | **Product**  **Size (bp)** | **Annealing Temperature (**°C**)** |
| --- | --- | --- | --- | --- | --- | --- | --- |
|  |  |  |  |  | **Antisense 5’-3’** |  |  |
| d17 | Ald | gi|110748949 | **Aldolase** | AGGACAATCGCAGAGCATAC | | 98 | 59.0 |
|  |  |  |  | CAAGGTCTCGTGGAATAGAATAAC | |  |  |
| u12 | Pglym78 | gi|66550890 | **Phosphoglyceromutase** | GCTGAAACTGCTGCTAAATATG | | 78 | 59.0 |
|  |  |  |  | CATAGGTGGAGGAGGTGTATC | |  |  |
| u9 | Idh | gi|110764717 | **Isocitrate dehydrogenase** | GGTTGGACCTGTTGTTGATG | | 177 | 59.0 |
|  |  |  |  | TTAATAGCCTCAGCACATTCTAC | |  |  |
| d9 | Hsp60 | gi|66547450 | **60 kDa heat shock protein** | AATGAAGAGGCAGGTGATGG | | 182 | 59.0 |
|  |  |  |  | TCAGGAGTTGTTACTGGCTTAC | |  |  |
| d6 | Hsp83 | gi|229892248 | **heat shock protein 90** | GCATTCTCAATTCATTGGTTATCC | | 89 | 59.0 |
|  |  |  |  | CTTCTTCCTCTTCTTCATCTTCAC | |  |  |
| u2 | Hsp68 | gi|229892210 | **heat shock protein cognate 4** | GCTGCTGCCATTGCTTATG | | 143 | 59.0 |
|  |  |  |  | TCTCCTGCTGTTGACTTGAC | |  |  |
| d12 | l(1)g0022 | gi|66513205 | **lethal (1) G0022** | CAAGATGATGTTACTGGAGATGG | | 113 | 59.0 |
|  |  |  |  | TCTGTAAGCACTCTAGGATGAAG | |  |  |
| d13 | Tcp-1η | gi|66540596 | **Tcp-1η** | TGATGCTATAATGGTTGTAAGACG | | 117 | 59.0 |
|  |  |  |  | CCTGCTATCACACGAGAATAATC | |  |  |
| u21 | tm2 | gi|66522386 | **Tropomyosin 2** | CCGATGAATCCAGCCGTATG | | 185 | 59.0 |
|  |  |  |  | ATCTTCAGCGACTTCCAACTC | |  |  |
| Control | Gapdh | gi|110760425 | **glyceraldehyde-3-phosphate dehydrogease** | GAT GCA CCC ATG TTT GTT TG | | 203 | 59.0 |
|  |  |  |  | TTT GCA GAA GGT GCA TCA AC | |  |  |

Spot number corresponds to the number of protein spots in Figure 3 and Table 2.
