# Supplementary material for: Proteomic Analysis of Honeybee (Apis mellifera L.) Pupae Head Development
Source: PLoS One. 2011 May 26;6(5):e20428. doi: 10.1371/journal.pone.0020428 (PMC3102718; doi:10.1371/journal.pone.0020428)
Supplement: Table S2 — Identification of differentially expressed proteins in honeybee (Apis mellifera L.) pupae head at different developmental stages. (DOC) [file pone.0020428.s002.doc]

**Table S2.** Identification of differentially expressed proteins in honeybee (*Apis mellifera* L.) pupae head at different developmental stages

| **Spot Number** | | | **Experimental *M*r(kDa)/p*I*** | | | | | | | **Theoretical *M*r(kDa)/p*I*** | | | | | **Sequence Coverage(%)** | | | | | **Matched/**  **Searched** | | | | **Score** | **Accession Number** | | | **Protein Name** | | **Mean±SD** | | | | | | | | | | | | | **One-way ANOVA**  **p value** | | |
| --- | --- | --- | --- | --- | --- | --- | --- | --- | --- | --- | --- | --- | --- | --- | --- | --- | --- | --- | --- | --- | --- | --- | --- | --- | --- | --- | --- | --- | --- | --- | --- | --- | --- | --- | --- | --- | --- | --- | --- | --- | --- | --- | --- | --- | --- |
|  |  |  |  |  |  |  |  |  |  |  |  |  |  |  |  |  |  |  |  |  |  |  |  |  |  |  |  |  |  | **Day13** | **Day15** | | | | **Day17** | | **Day19** | **Day20** | | | | |  |  |  |
| [**Carbohydrate**](app:ds:carbohydrate) **metabolism and** [**energy**](app:ds:energy) **production** | | | | | | | | | | | | | | | | | | | | | | | | | | | | | | | | | | | | | | | | | | | | | |
| d15 | | | | | 48.74/5.57 | | | | 49.42/5.70 | | | | | | 58 | | | | | 23/57 | | | 232 | | gi|110763974 | | | **aldehyde dehydrogenase (NAD, EC.1.2.1.3)** | 1865±381^a^ | | 1782±31^a^ | | | | 1637±40^a^ | | 953±25^b^ | | 658±91^c^ | | | | | | 0.031 |
| d14 | | | | | 60.44/6.08 | | | | 58.32/6.22 | | | | | | 37 | | | | | 24/69 | | | 114 | | gi|66533395 | | | **ATP phosphohydrolase (EC.3.6.1.3)** | 590±104^a^ | | 441±94^b^ | | | | 100±20^c^ | | 535±41^a^ | | 428±45^b^ | | | | | | 0.012 |
| u5 | | | | | 58.37/6.41 | | | | 55.12/7.55 | | | | | | 46 | | | | | 21/96 | | | 157 | | gi|66526646 | | | **Dihydrolipoyl dehydrogenase (EC.1.8.1.4)** | 1257±36^a^ | | 576±16^b^ | | | | 773±15^b^ | | 1424±19^a^ | | 1461±21^a^ | | | | | | 0.006 |
| u6 | | | | | 48.00/4.96 | | | | 55.10/5.25 | | | | | | 53 | | | | | 23/36 | | | 215 | | gi|110762902 | | | **ATP synthase (atpsyn, EC.3.6.3.14)** | 1306±171^c^ | | 1713±80^c^ | | | | 1356±16^c^ | | 3834±75^b^ | | 5808±99^a^ | | | | | | 0.010 |
| u9 | | | | | 44.41/6.86 | | | | 47.02/6.87 | | | | | | 75 | | | | | 30/83 | | | 272 | | gi|110764717 | | | **Isocitrate dehydrogenase (idh, EC.1.1.1.42)** | 989±397^c^ | | 500±16^d^ | | | | 1386±85^c^ | | 2314±138^a^ | | 1869±61^b^ | | | | | | 0.002 |
| d19 | | | | | 42.37/5.66 | | | | 40.33/5.66 | | | | | | 66 | | | | | 29/102 | | | 232 | | gi|58585146 | | | **Arginine kinase (argk, EC.2.7.3.3)** | 3500±132^c^ | | 3438±90^c^ | | | | 5278±50^a^ | | 4956±53^a^ | | 4430±158^b^ | | | | | | 0.003 |
| d17 | | | | | 44.22/6.99 | | | | 39.98/7.57 | | | | | | 33 | | | | | 11/44 | | | 106 | | gi|110748949 | | | **Aldolase (ald, EC.4.1.2.13)** | 338±94^c^ | | 1766±108^a^ | | | | 476±23^c^ | | 952±41^b^ | | 827±45^b^ | | | | | | 0.034 |
| d20 | | | | | 41.66/8.24 | | | | 36.15/8.11 | | | | | | 36 | | | | | 14/69 | | | 86 | | gi|48142692 | | | **Glyceraldehyde-3-phosphate dehydrogenase 2 (gapdh2, EC.1.2.1.12)** | 1060±294^a^ | | 970±318^a^ | | | | 940±38^a^ | | 0±0^b^ | | 0±0^b^ | | | | | | 0.029 |
| d22 | | | | | 41.00/5.65 | | | | 40.33/5.66 | | | | | | 49 | | | | | 18/42 | | | 166 | | gi|58585146 | | | **arginine kinase (argk, EC.2.7.3.3)** | 2071±115^a^ | | 770±37^b^ | | | | 304±29^a^ | | 0±0^d^ | | 0±0^d^ | | | | | | 0.012 |
| d24 | | | | | 40.15/5.95 | | | | 36.88/6.07 | | | | | | 29 | | | | | 7/14 | | | 108 | | gi|66530373 | | | **CG3609** | 1634±22^a^ | | 1200±131^b^ | | | | 1145±33^b^ | | 896±182^c^ | | 589±74^d^ | | | | | | 0.009 |
| u12 | | | | | 23.34/6.98 | | | | 35.40/9.36 | | | | | | 60 | | | | | 25/91 | | | 169 | | gi|66550890 | | | **Phosphoglyceromutase (pglym78, EC.5.4.2.1)** | 1751±100^c^ | | 1248±71^d^ | | | | 2004±25^c^ | | 2509±353^b^ | | 3761±82^a^ | | | | | | 0.001 |
| u17 | | | | | 11.15/7.81 | | | | 11.40/8.01 | | | | | | 75 | | | | | 10/43 | | | 143 | | gi|66547447 | | | **ATP phosphohydrolase (EC.3.6.1.3)** | 2246±670^c^ | | 454±56^d^ | | | | 2716±49^b^ | | 2907±730^b^ | | 5577±66^a^ | | | | | | 0.000 |
| **Development** | | | | | | | | | | | | | | | | | | | | | | | | | | | | | | | | | | | | | | | | | | | | | |
| u1 | | | | | 96.86/5.22 | | | 89.47/5.18 | | | | | | | | 65 | | | | 57/133 | | 316 | | | gi|66534286 | | | **TER94** | 897±255^b^ | | 459±99^c^ | | | 603±41^c^ | | | 1396±60^a^ | | 977±94^b^ | | | | | 0.035 | |
| d10 | | | | | 63.66/5.98 | | | 58.79/6.06 | | | | | | | | 52 | | | | 26/126 | | 171 | | | gi|66549818 | | | **singed (sn)** | 502±11^c^ | | 958±193^b^ | | | 1251±30^a^ | | | 715±18^c^ | | 609±46^c^ | | | | | 0.003 | |
| d11 | | | | | 63.26/6.05 | | | 58.79/6.06 | | | | | | | | 57 | | | | 28/107 | | 243 | | | gi|66549818 | | | **singed (sn)** | 1016±98^c^ | | 1556±357^a^ | | | 1320±16^b^ | | | 620±87^d^ | | 633±44^d^ | | | | | 0.009 | |
| u3 | | | | | 60.02/4.49 | | | 47.49/4.45 | | | | | | | | 45 | | | | 15/23 | | 193 | | | gi|66545506 | | | **Calreticulin (crc)** | 2437±203^c^ | | 4528±333^b^ | | | 4925±73^b^ | | | 8012±114^a^ | | 8388±465^a^ | | | | | 0.010 | |
| d13 | | | | | 60.62/6.01 | | | 60.41/6.03 | | | | | | | | 44 | | | | 25/112 | | 108 | | | gi|66540596 | | | **Tcp-1η (EC.3.6.1.3)** | 1557±396^a^ | | 164±124^c^ | | | 341±58^b^ | | | 254±19^b^ | | 341±61^b^ | | | | | 0.000 | |
| d12 | | | | | 63.53/6.12 | | | 44.21/6.58 | | | | | | | | 49 | | | | 16/75 | | 87 | | | gi|66513205 | | | **lethal (1) G0022 [l(l)g0022]** | 1512±42^a^ | | 1082±311^b^ | | | 664±23^c^ | | | 746±55^c^ | | 988±98^b^ | | | | | 0.028 | |
| u10 | | | | | 36.15/4.61 | | | 36.14/4.61 | | | | | | | | 37 | | | | 12/28 | | 133 | | | gi|66530530 | | | **Annexin IX (AnnIX)** | 1249±315^d^ | | 1058±59^d^ | | | 1928±16^c^ | | | 4505±149^b^ | | 5340±55^a^ | | | | | 0.003 | |
| u11 | | | | | 33.22/5.28 | | | 33.61/5.38 | | | | | | | | 76 | | | | 22/108 | | 165 | | | gi|66519254 | | | **Soluble NSF attachment protein (snap)** | 1338±57^b^ | | 951±45^c^ | | | 989±34^c^ | | | 2032±13^a^ | | 2173±29^a^ | | | | | 0.000 | |
| u13 | | | | | 23.04/4.69 | | | 28.17/4.79 | | | | | | | | 72 | | | | 27/81 | | 177 | | | gi|66517155 | | | **14-3-3 protein zeta (14-3-3 zeta)** | 1785±419^c^ | | 1620±123^c^ | | | 3734±49^b^ | | | 3766±78^b^ | | 5759±31^a^ | | | | | 0.001 | |
| u14 | 21.76/4.44 | | | | | 30.17/4.89 | | | | | 33 | | | | | | 10/64 | | | | 92 | | | | gi|66508940 | **CG6459** | | | 604±124^d^ | | | | 849±87^d^ | | 2105±37^c^ | 3410±158^b^ | | | | | | 3774±167^a^ | | 0.004 | |
| d30 | | | | | 21.05/6.62 | | | 24.86/6.96 | | | | | | | | 26 | | | | 6/6 | | 103 | | | gi|48097366 | | | **ran** | 479±75^d^ | | 4348±152^a^ | | | 1836±60^b^ | | | 1357±41^b^ | | 944±65^c^ | | | | | 0.000 | |
| d31 | | | | | 20.69/4.50 | | | 19.83/4.57 | | | | | | | | 56 | | | | 17/59 | | 138 | | | gi|66515987 | | | **Translationally controlled tumor protein (Tctp)** | 1931±64^a^ | | 1286±170^c^ | | | 1577±34^b^ | | | 1400±93^b^ | | 1469±158^b^ | | | | | 0.033 | |
| **Cytoskeleton** | | | | | | | | | | | | | | | | | | | | | | | | | | | | | | | | | | | | | | | | | | | | | |
| u8 | | | | | 44.49/5.21 | | | | 42.20/5.30 | | | | | | | 35 | | | | 11/21 | | 123 | | | gi|66509769 | | | **Actin-87E (Act87E)** | 8259±147^d^ | | 9908±186^c^ | | | 13800±49^b^ | | | 32140±232^a^ | | | 32230±557^a^ | | | | 0.000 | |
| u21 | | | | | 37.49/4.68 | | | | 32.25/4.73 | | | | | | | 43 | | | | 14/55 | | 104 | | | gi|66522386 | | | **Tropomyosin 2 (tm2)** | 0±0^c^ | | 0±0^c^ | | | 0±0^c^ | | | 803±26^b^ | | | 1848±35^a^ | | | | 0.000 | |
| d25 | | | | | 34.66/5.08 | | | | 50.60/4.75 | | | | | | | 52 | | | | 25/76 | | 176 | | | gi|48095525 | | | **Tubulin at 56D (tub56d)** | 792±88^d^ | | 3272±445^a^ | | | 1346±35^c^ | | | 1495±244^c^ | | | 2061±50^b^ | | | | 0.000 | |
| d26 | | | | | 24.08/7.16 | | | | 31.28/8.44 | | | | | | | 39 | | | | 6/13 | | 85 | | | gi|110764441 | | | **Ccp84Ad** | 3596±209^a^ | | 2675±93^b^ | | | 2102±103^c^ | | | 2586±50^b^ | | | 1704±76^d^ | | | | 0.049 | |
| d27 | | | | | 24.46/7.57 | | | | 31.28/8.44 | | | | | | | 52 | | | | 10/97 | | 91 | | | gi|110764441 | | | **Ccp84Ad** | 4575±144^c^ | | 5497±158^b^ | | | 9223±145^a^ | | | 4074±180^c^ | | | 2086±199^d^ | | | | 0.003 | |
| u23 | | | | | 23.48/4.67 | | | | 23.53/4.78 | | | | | | | 48 | | | | 6/10 | | 91 | | | [gi|66555437](http://www.ncbi.nlm.nih.gov/blast/Blast.cgi?ALIGNMENTS=50&ALIGNMENT_VIEW=Pairwise&AUTO_FORMAT=Semiauto&CDD_SEARCH=on&CLIENT=web&COMPOSITION_BASED_STATISTICS=on&DATABASE=nr&DESCRIPTIONS=100&ENTREZ_QUERY=(none)&EXPECT=10&FILTER=L&FORMAT_BLOCK_ON_RESPAGE=None&FORMAT_OBJECT=Alignment&FORMAT_TYPE=HTML&GAPCOSTS=11+1&I_THRESH=0.001&LAYOUT=TwoWindows&MATRIX_NAME=BLOSUM62&NCBI_GI=on&PAGE=Proteins&PROGRAM=blastp&QUERY=MADKEKKKKTKKKEEAAPAPPPPEPEPEPEKPPTPAPSTPKESGSTRASSRGSRKAKRAGSSVFSMFTQKQVAEFKEAFQLMDQDKDGIIGKNDLRATFDNVGRLVTDKELDDMLNEAPAPINFTQLLNLFASRMSGSGQDDDETVIAAFSTFDVNGKIDGERLRHALMTYGDKFTAKEVNDAYDNMYIDDKGFIDTQSLIAMLTGQEDEDEE&SERVICE=plain&SET_DEFAULTS.x=9&SET_DEFAULTS.y=5&SHOW_OVERVIEW=on&WORD_SIZE=3&END_OF_HTTPGET=Yes) | | | **Myosin regulatory light chain 2 (mlc2)** | 0±0^b^ | | 0±0^b^ | | | 0±0^b^ | | | 0±0^b^ | | | 1861±89^a^ | | | | 0.000 | |
| d29 | | | | | 21.63/6.58 | | | | 21.56/6.82 | | | | | | | 60 | | | | 11/65 | | 130 | | | gi|110764439 | | | **Cuticular protein 64Aa** | 2124±97^a^ | | 0±0^b^ | | | 0±0^b^ | | | 0±0^b^ | | | 0±0^b^ | | | | 0.000 | |
| d32 | | | | | 17.7/5.42 | | | | 17.05/6.17 | | | | | | | 70 | | | | 12/60 | | 139 | | | gi|110751158 | | | **Cofilin/actin-depolymerizing factor homolog (tsr)** | 3001±125^b^ | | 2674±154^c^ | | | 3546±72^a^ | | | 2265±58^c^ | | | 1271±45^d^ | | | | 0.010 | |
| u15 | | | | | 16.8/5.77 | | | | 17.05/6.17 | | | | | | | 56 | | | | 8/10 | | 156 | | | gi|110751158 | | | **Cofilin/actin-depolymerizing factor homolog (tsr)** | 3448±116^c^ | | 3187±276^d^ | | | 3299±27^d^ | | | 4096±443^b^ | | | 6099±155^a^ | | | | 0.023 | |
| **Protein biosynthesis** | | | | | | | | | | | | | | | | | | | | | | | | | | | | | | | | | | | | | | | | | | | | | |
| d1 | | | | 112.00/6.19 | | | | 111.92/6.43 | | | | | | | 10 | | | | 8/12 | | | 87 | | | gi|156637469 | | | **hexamerin 110 (Lsp2)** | 11540±100^a^ | | | 10089±79^b^ | | 2611±554^c^ | | | 776±93^d^ | | | | 364±62^e^ | | | 0.000 | |
| d2 | | | | 111.80/6.25 | | | | 111.92/6.43 | | | | | | | 21 | | | | 14/29 | | | 114 | | | gi|156637469 | | | **hexamerin 110 (Lsp2)** | 21803±594^a^ | | | 12126±89^b^ | | 6804±348^c^ | | | 1316±76^d^ | | | | 476±91^e^ | | | 0.000 | |
| d3 | | | | 111.80/6.4 | | | | 111.92/6.43 | | | | | | | 24 | | | | 16/32 | | | 134 | | | gi|156637469 | | | **hexamerin 110 (Lsp2)** | 23670±348^a^ | | | 16957±215^b^ | | 6729±42^c^ | | | 1858±31^d^ | | | | 321±18^e^ | | | 0.000 | |
| d4 | | | | 111.80/6.5 | | | | 111.92/6.43 | | | | | | | 17 | | | | 12/26 | | | 96 | | | gi|156637469 | | | **hexamerin 110 (Lsp2)** | 12010±379^a^ | | | 11090±470^b^ | | 3206±92^c^ | | | 854±46^d^ | | | | 361±11^e^ | | | 0.000 | |
| d5 | | | | 99.12/6.55 | | | | 111.92/6.43 | | | | | | | 16 | | | | 11/25 | | | 85 | | | gi|156637469 | | | **hexamerin 110 (Lsp2)** | 1815±217^c^ | | | 2833±309^b^ | | 4484±189^a^ | | | 1324±52^d^ | | | | 222±64^e^ | | | 0.006 | |
| d7 | | | | 93.02/6.13 | | | | 111.92/6.43 | | | | | | | 14 | | | | 10/17 | | | 90 | | | gi|156637469 | | | **hexamerin 110 (Lsp2)** | 453±98^c^ | | | 2847±89^a^ | | 2118±411^b^ | | | 240±10^d^ | | | | 113±5^d^ | | | 0.014 | |
| u7 | | | | 45.66/6.86 | | | | 53.01/8.16 | | | | | | | 58 | | | | 35/137 | | | 170 | | | gi|66518848 | | | **Elongation factor Tu mitochondrial (eftum)** | 1817±45^b^ | | | 635±12^d^ | | 1609±234^c^ | | | 2601±22^a^ | | | | 1797±47^b^ | | | 0.000 | |
| **Protein folding** | | | | | | | | | | | | | | | | | | | | | | | | | | | | | | | | | | | | | | | | | | | | | |
| d6 | | | | 91.36/4.96 | | | | 83.79/4.98 | | | | | | 39 | | | | | 33/70 | | | 227 | | | gi|229892248 | | | **heat shock protein 90 (hsp83)** | 1174±146^b^ | | | 712±60^c^ | | | 1349±106^a^ | | 1208±66^a^ | | | | | 1052±29^b^ | | 0.028 | |
| d8 | | | | 78.84/5.12 | | | | 72.88/5.29 | | | | | | 32 | | | | | 24/65 | | | 133 | | | gi|229892214 | | | **heat shock protein cognate 3 (hsc70-3)** | 2852±402^a^ | | | 1340±135^d^ | | | 1332±63^d^ | | 2076±552^c^ | | | | | 2417±174^b^ | | 0.011 | |
| u2 | | | | 75.01/5.43 | | | | 71.38/5.43 | | | | | | 40 | | | | | 22/34 | | | 205 | | | gi|229892210 | | | **heat shock protein cognate 4 (hsc70-4)** | 2458±66^b^ | | | 2597±78^a^ | | | 1578±64^d^ | | 2640±58^a^ | | | | | 2212±52^c^ | | 0.044 | |
| d9 | | | | 60.95/5.27 | | | | 60.55/5.64 | | | | | | 34 | | | | | 17/34 | | | 134 | | | gi|66547450 | | | **60 kDa heat shock protein (hsp60)** | 2956±443^a^ | | | 2290±501^b^ | | | 1190±99^c^ | | 1285±348^c^ | | | | | 1034±53^c^ | | 0.027 | |
| u4 | | | | 63.44/6.37 | | | | 55.78/6.24 | | | | | | 46 | | | | | 23/107 | | | 89 | | | gi|110756123 | | | **Hsp70/Hsp90 organizing protein homolog (hop)** | 744±56^b^ | | | 522±19^c^ | | | 571±16^c^ | | 1039±34^a^ | | | | | 1298±12^a^ | | 0.001 | |
| d28 | | | | 22.12/5.24 | | | | 25.61/6.01 | | | | | | 29 | | | | | 8/14 | | | 120 | | | [gi|110757651](http://www.ncbi.nlm.nih.gov/blast/Blast.cgi?ALIGNMENTS=50&ALIGNMENT_VIEW=Pairwise&AUTO_FORMAT=Semiauto&CDD_SEARCH=on&CLIENT=web&COMPOSITION_BASED_STATISTICS=on&DATABASE=nr&DESCRIPTIONS=100&ENTREZ_QUERY=(none)&EXPECT=10&FILTER=L&FORMAT_BLOCK_ON_RESPAGE=None&FORMAT_OBJECT=Alignment&FORMAT_TYPE=HTML&GAPCOSTS=11+1&I_THRESH=0.001&LAYOUT=TwoWindows&MATRIX_NAME=BLOSUM62&NCBI_GI=on&PAGE=Proteins&PROGRAM=blastp&QUERY=MADSGIKRNIPIKLGDFSVIDTEFSNIRERFDAEMRKMEDEMSRFRSELMNRESNNFFKSTTSRHHTSTSEHRTSTTSKSEGWDKVDPAAPPTRSAFDSFKSTTTQSTQNSSLSPPHDSAWLDGLNSPLIQDEGDSKCLKLRFDVSQYTPEEIVVKTVDNKLLVHAKHEEKTESKSVYREYNREFLLPKGTNPESIKSSLSKDGVLTVEAPLPAIGTGEKLIPIAHQ&SERVICE=plain&SET_DEFAULTS.x=9&SET_DEFAULTS.y=5&SHOW_OVERVIEW=on&WORD_SIZE=3&END_OF_HTTPGET=Yes) | | | **small heat shock protein (hsp20)** | 10633±56^a^ | | | 8574±120^b^ | | | 6531±56^c^ | | 4297±123^d^ | | | | | 1610±89^e^ | | 0.030 | |
| u22 | | | | 21.89/5.31 | | | | 25.61/6.01 | | | | | | 44 | | | | | 14/44 | | | 130 | | | gi|110757651 | | | **small heat shock protein (hsp20)** | 0±0^c^ | | | 0±0^c^ | | | 0±0^c^ | | 2666±143^b^ | | | | | 2826±130^a^ | | 0.000 | |
| **Transporter** | | | | | | | | | | | | | | | | | | | | | | | | | | | | | | | | | | | | | | | | | | | | | |
| d16 | | | 45.51/6.13 | | | | | 51.84/5.47 | | | | | 53 | | | | | 24/108 | | | | 178 | | | gi|66499186 | | | **FK506-binding protein FKBP59 (FKBP59)** | 1551±149^a^ | | | 585±50^d^ | | | 862±55^c^ | | 1185±247^b^ | | | | | 608±18^d^ | | 0.002 | |
| u19 | | | 21.41/4.76 | | | | | 26.60/4.93 | | | | | 38 | | | | | 9/17 | | | | 131 | | | [gi|66548280](http://www.ncbi.nlm.nih.gov/blast/Blast.cgi?ALIGNMENTS=50&ALIGNMENT_VIEW=Pairwise&AUTO_FORMAT=Semiauto&CDD_SEARCH=on&CLIENT=web&COMPOSITION_BASED_STATISTICS=on&DATABASE=nr&DESCRIPTIONS=100&ENTREZ_QUERY=(none)&EXPECT=10&FILTER=L&FORMAT_BLOCK_ON_RESPAGE=None&FORMAT_OBJECT=Alignment&FORMAT_TYPE=HTML&GAPCOSTS=11+1&I_THRESH=0.001&LAYOUT=TwoWindows&MATRIX_NAME=BLOSUM62&NCBI_GI=on&PAGE=Proteins&PROGRAM=blastp&QUERY=MDRWAGKVAVVTGASAGIGAAIVKQLLTHGMVVAGLARRVEKIKELEQGLEECTGKLYAVECDVSKEESVIAAFAWVQENLGPANVLINNAGITKESSLIDGNLEDWRSVFDVNVFGLCLCTKEAIRMMRETGGEGVIININSLAGERVPFIPGFSVYPASKRAIAALAQTLRHELTGTQIRVTGISPGLVATELMVSYSTYSEEALASFPTLDPEDVATAAIYILSCAPHVVVQDIILRPLGESW&SERVICE=plain&SET_DEFAULTS.x=9&SET_DEFAULTS.y=5&SHOW_OVERVIEW=on&WORD_SIZE=3&END_OF_HTTPGET=Yes) | | | **antdh (antdh)** | 0±0^e^ | | | 373±26^d^ | | | 1256±26^c^ | | 2657±202^a^ | | | | | 2184±35^b^ | | 0.000 | |
| d34 | | | 13.87/5.38 | | | | | 15.59/5.71 | | | | | 42 | | | | | 6/9 | | | | 106 | | | gi|94158822 | | | **odorant binding protein 14 (Obp22a)** | 7634±298^a^ | | | 3762±231^c^ | | | 4208±38^b^ | | 4102±299^b^ | | | | | 2234±468^d^ | | 0.035 | |
| d35 | | | 12.19/5.52 | | | | | 15.49/6.37 | | | | | 57 | | | | | 13/50 | | | | 114 | | | gi|94158810 | | | **odorant binding protein 13 (Obp18a)** | 1997±68^a^ | | | 665±105^d^ | | | 1713±67^b^ | | 618±30^d^ | | | | | 1078±72^c^ | | 0.025 | |
| [**Amino acid**](app:ds:amino acid) **metabolism** | | | | | | | | | | | | | | | | | | | | | | | | | | | | | | | | | | | | | | | | | | | | | |
| d23 | | 40.49/6.29 | | | | | | 41.81/6.42 | | | | | 47 | | | | | 18/65 | | | 146 | | | | gi|283436154 | | | **glutamine synthetase (Gs, EC.6.3.1.2)** | 461±47^b^ | | | 338±29^b^ | | | 1107±20^a^ | | 485±71^b^ | | | | | 906±31^a^ | | 0.003 | |
| d33 | | 14.47/5.05 | | | | | | 26.82/4.83 | | | | | 56 | | | | | 12/42 | | | 111 | | | | gi|66541426 | | | **Proteasome subunit alpha type 5 (prosma5, EC.3.4.25.1)** | 1988±32^a^ | | | 435±128^d^ | | | 1267±11^c^ | | 1627±85^b^ | | | | | 1471±48^c^ | | 0.023 | |
| d36 | | 21.75/6.12 | | | | | | 30.69/7.03 | | | | | 43 | | | | | 11/24 | | | 139 | | | | gi|66519842 | | | **Proteasome 2 subunit (prosbeta2, EC.3.4.25.1)** | 0±0^d^ | | | 6368±404^a^ | | | 4911±305^b^ | | 6284±248^a^ | | | | | 3212±137^c^ | | 0.014 | |
| **Nucleotide metabolism** | | | | | | | | | | | | | | | | | | | | | | | | | | | | | | | | | | | | | | | | | | | | | |
| d18 | | | 42.50/5.33 | | | | 43.73/5.60 | | | | | 24 | | | | | | 8/15 | | | 86 | | | | gi|66513629 | | **beta-ureidopropionase (pyd3, EC.3.5.1.6 )** | | 2964±62^a^ | | | | 2431±143^b^ | | 2161±191^c^ | | 1389±63^d^ | | | | | 859±77^e^ | | 0.028 | |
| d21 | | | 42.48/5.56 | | | | 43.73/5.60 | | | | | 35 | | | | | | 12/19 | | | 133 | | | | gi|66513629 | | **beta-ureidopropionase (pyd3, EC.3.5.1.6 ) ()** | | 10898±553^a^ | | | | 5889±578^b^ | | 5134±579^c^ | | 3929±338^d^ | | | | | 979±51^e^ | | 0.035 | |
| **Antioxidant system** | | | | | | | | | | | | | | | | | | | | | | | | | | | | | | | | | | | | | | | | | | | | | |
| u20 | | 19.38/5.23 | | | | | | 23.40/5.64 | | | 54 | | | | | | | 14/59 | | | 118 | | | | gi|110756698 | | **Phospholipid hydroperoxide glutathione peroxidase (phgpx, EC.1.11.1.12)** | | 0±0^c^ | | | | 0±0^c^ | | 1067±49^a^ | | 1202±59^a^ | | | | | 726±23^b^ | | 0.025 | |
| [**fatty acid**](app:ds:fatty acid) **metabolism** | | | | | | | | | | | | | | | | | | | | | | | | | | | | | | | | | | | | | | | | | | | | | |
| u16 | 13.68/6.44 | | | | | | 15.14/6.37 | | | | 85 | | | | | | | 17/60 | | | 152 | | | | gi|58585202 | **fatty acid binding protein (rfabp)** | | | 200±20^e^ | | | | 2231±229^c^ | | 1960±177^d^ | | 4956±188^a^ | | | | | 3585±57^b^ | | 0.007 | |
| **Unknown function** | | | | | | | | | | | | | | | | | | | | | | | | | | | | | | | | | | | | | | | | | | | | | |
| u18 | 85.38/4.64 | | | | | 79.17/4.96 | | | | | 34 | | | | | | 29/99 | | | | 154 | | | | gi|48132776 | **CG7802** | | | 0±0^d^ | | | | 1740±208^b^ | | 2144±173^a^ | 2223±109^a^ | | | | | | 486±24^c^ | | 0.006 | |

All identified proteins are from *Apis mellifera*. Means followed by different superscript (a, b, c, d, e) are significantly different (p<0.05). Spot number corresponds to the number of protein spots in Figure 3. Theoretical molecular weight (*M*r) and isoelectric point (p*I*) of the identified proteins were retrieved from the protein database of NCBInr. Experimental *M*r and p*I* were calculated using the PDQuest Software and internal standard molecular mass markers. Sequence coverage is the ratio of the number of amino acids in every peptide that matches with the mass spectrum divided by the total number of amino acids in the protein sequence. Matched peptide is the number of paring an experimental fragmentation spectrum to a theoretical segment of protein and searched is the total searched peptide. Mascot score is searched against the database NCBInr. Mean±SD is the mean value of protein amount ± standard deviation. Protein name is given when proteins were identified by MALDI-TOF/MS. Accession number is the unique number given to mark the entry of a protein in the database NCBInr.
